# Supplementary material for: The Characteristic Function of Blood-Derived Exosomes and Exosomal circRNAs Isolated from Dairy Cattle during the Dry Period and Mid-Lactation
Source: Int J Mol Sci. 2023 Jul 29;24(15):12166. doi: 10.3390/ijms241512166 (PMC10419012; doi:10.3390/ijms241512166)
Supplement: Supplementary file 1 [file ijms-24-12166-s001.zip › Table S1.pdf]

Table S1A. RT-qPCR primers used to quantify the expression of differentially expressed genes in D30 and D250 exosomes

| Gene_name | Forward(5'→3') | Reverse(5'→3') | Amplicon size(bp) |
|---|---|---|---|
| ASRGL1 | GAAAGAAGCAGAAAACGC | CGTAACCTCCAGATCCTACA | 202 |
| EIF3E | CTTCTTGGTGGCTTGTCT | AGCCTTGCATTTCTAATC | 171 |
| RPSA | GTGGGTCTGATGTGGTGG | GAGCAGCCGTGAACTCTG | 214 |
| MRPS6 | TGGTGAGGAGCCTGGAGA | CCTGGGTCAGAGGGTGTT | 198 |
| RPS14 | AAGGGGAAGGAAAAGAAG | AGTTACACGGCAGATGGT | 159 |
| EZR | TGTACGAGCCGGTGAACT | GGATGATGTCGTTGTGGG | 222 |
| HNRNPA2B1 | GATGGCTATAATGGGTATG | AAGCTCAGTATCTGCTTCTC | 223 |
| RIPOR2 | TGCTCTACTACTGTGAAGCCCTAA | ATATGCCAGCCGCCCATC | 194 |
| PARN | ACAAGTGCCGATTGCTGT | GTGCTGGTGGCTGTGAGA | 222 |
| PICALM | GCAAGTACATGGGGAGGG | GTTTGGCAACGGGAAGAC | 196 |
| HDAC9 | GTTCTCCACCAAACTCCAA | TCTTCCATAGGCTCACCAG | 236 |
| RPL3 | TTTGTCCACTACGGTGAG | CAATTCGGTCCTTCTTAA | 235 |
| FCGR3A | TTCCACATTCCAGAAGCA | GCAAACAGGACTCCCATC | 191 |
| cel-miR-39 | GGGTCACCGGGTGTAAATC | CAGTGCGTGTCGTGGAGT | |

Table S1B. PCR primers used to amplify specific circular RNAs

| CircRNA | Forward(5'→3') | Reverse(5'→3') | Amplicon size(bp) |
|---|---|---|---|
| circ_AFF3 | AGAGTCATCTTGTGGGTGTTCC | GCCATTGCCGCTGTGCTT | 365 |
| circ_CHD1 | GTCGCCAAGCAACTGTCA | TTTCCTCTGTCTCCCAAG | 312 |
| circ_ITGA6 | AACTGCGTCCCATTCCCATAA | ATGGAAGAAACCCTCTGG | 283 |
| circ_PRMT2 | GGTGGAGGCCAGTGAGAT | CACTATGCTCAGCCCAGAG | 317 |
| circ_FAM120A | TCTGGCTTCCTTTCATTG | CATGAGGTTGCCAAGCAG | 259 |
| circ_RHBDD1 | TCGGTTTCTCAGGGAGTATTG | ACGTTGCCAGGGTGACAGG | 315 |
| circ_ATL3 | CGATGTGGTGGTGGTGTC | TCTCGGATGTGGTCCTGTA | 358 |
| circ_TMTC2 | AGGAGCAAGGAGTCACGG | TCAGGCGGAAAGAAAGAGT | 358 |
| circ_DENND4A | TGAAGTCAAGCCCATCTA | ATTCAGAAAGGGCAAGAA | 283 |
| circ_RRAS2 | GAGGGTTTCCTTTTGGTC | CTCTTGTCCCGCTGTATC | 261 |
